# Listening to voices: understanding and self-management of auditory verbal hallucinations in young adults

**DOI:** 10.1192/bjo.2021.107

**Published:** 2021-06-18

**Authors:** Peter Denno, Stephanie Wallis, Jonathan Ives, Stephen Wood, Matthew Broome, Pavan Mallikarjun, Femi Oyebode, Rachel Upthegrove, Kimberly Caldwell

**Affiliations:** 1 University of Birmingham, Medical School; 2 University of Bristol; 3 Orygen; 4 University of Birmingham; 5 Institute for Mental Health, School of Psychology, University of Birmingham

## Abstract

**Aims:**

Auditory Verbal Hallucinations (AVH) are a hallmark of psychosis, but affect many other clinical populations. Patients’ understanding and self-management of AVH may differ between diagnostic groups, change over time, and influence clinical outcomes.

We aimed to explore patients’ understanding and self-management of AVH in a young adult clinical population.

**Method:**

35 participants reporting frequent AVH were purposively sampled from a youth mental health service, to capture experiences across psychosis and non-psychosis diagnoses. Diary and photo-elicitation methodologies were used – participants were asked to complete diaries documenting experiences of AVH, and to take photographs representing these experiences. In-depth, unstructured interviews were held, using participant-produced materials as a topic guide. Conventional content analysis was conducted, deriving results from the data in the form of themes.

**Result:**

Three themes emerged:

1. Searching for answers, forming identities – voice-hearers sought to explain their experiences, resulting in the construction of identities for voices, and descriptions of relationships with them. These identities were drawn from participants’ life-stories (e.g., reflecting trauma), and belief-systems (e.g., reflecting supernatural beliefs, or mental illness). Some described this process as active / volitional. Participants described re-defining their own identities in relation to those constructed for AVH (e.g. as diseased, 'chosen', or persecuted), others considered AVH explicitly as aspects of, or changes in, their personality.
2. Coping strategies and goals – patients’ self-management strategies were diverse, reflecting the diverse negative experiences of AVH. Strategies were related to a smaller number of goals, e.g. distraction, soothing overwhelming emotions, 'reality-checking', and retaining agency.
3. Outlook – participants formed an overall outlook reflecting their self-efficacy in managing AVH. Resignation and hopelessness in connection with disabling AVH are contrasted with outlooks of “acceptance” or integration, which were described as positive, ideal, or mature.

**Conclusion:**

Trans-diagnostic commonalities in understanding and self-management of AVH are highlighted - answer-seeking and identity-formation processes; a diversity of coping strategies and goals; and striving to accept the symptom. Descriptions of “voices-as-self”, and dysfunctional relationships with AVH, could represent specific features of voice-hearing in personality disorder, whereas certain supernatural/paranormal identities and explanations were clearly delusional. However, no aspect of identity-formation was completely unique to psychosis or non-psychosis diagnostic groups. The identity-formation process, coping strategies, and outlooks can be seen as a framework both for individual therapies and further research.

